# Supplementary material for: Molecular and functional characterization of telomeric repeat-containing RNAs in Chinese hamster ovary cells
Source: Nucleic Acids Res. 2026 Jul 27;54(14):gkag739. doi: 10.1093/nar/gkag739 (PMC13403905; doi:10.1093/nar/gkag739)

Supplementary Figures

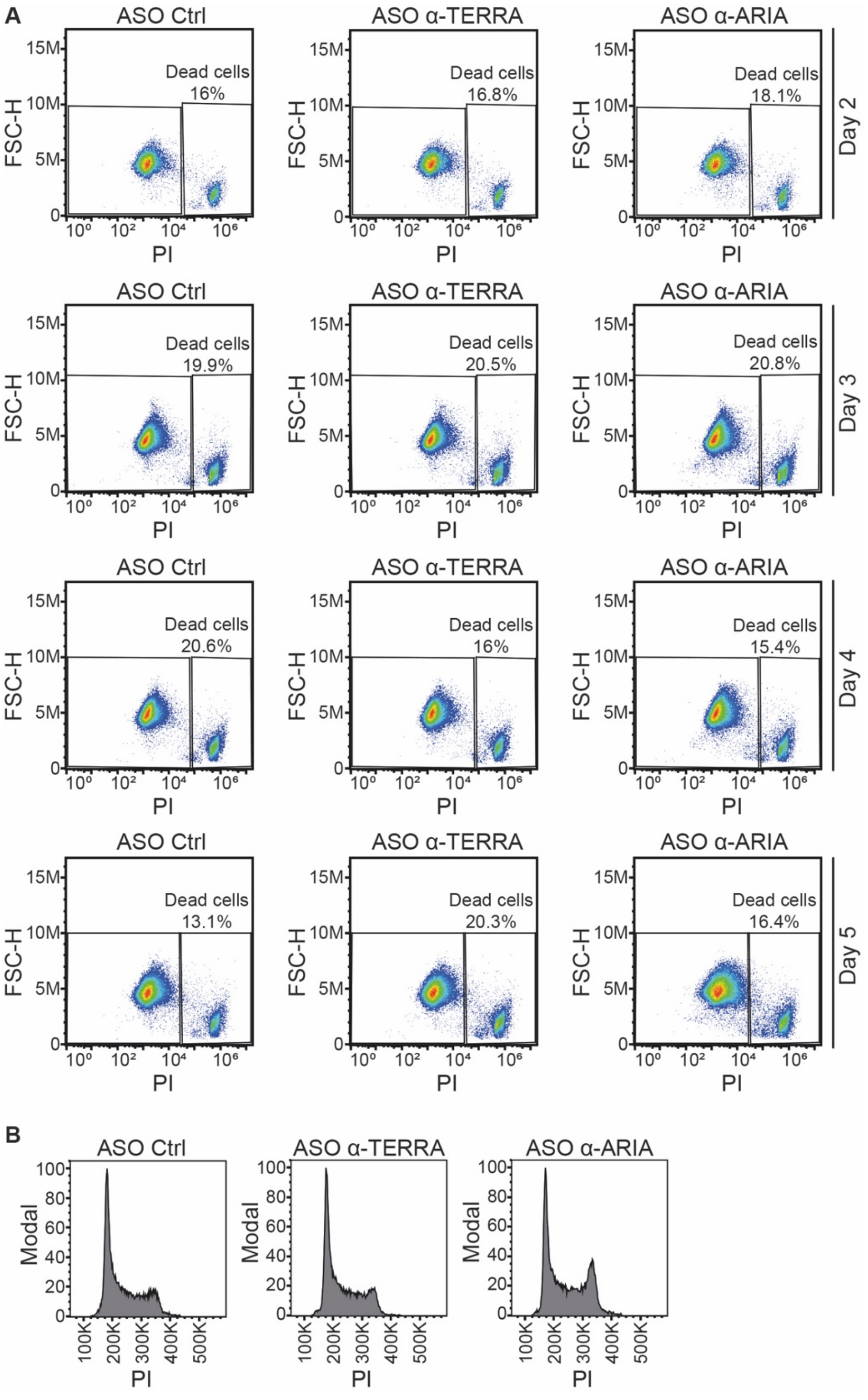

Supplementary Figure S1: Cell death and cell cycle analysis of TERRA- and ARIA-depleted CHO cells. (**A**) Representative fluorescence-activated cell sorting (FACS) analysis of ASO-transfected cells stained with propidium iodide (PI) in the absence of permeabilization and fixation. The fraction (%) of PI-positive, dead cells is indicated. FSC-H: Forward Scatter-Height. (**B**) Representative FACS analysis of ASO-transfected cells stained with PI after permeabilization and fixation using ethanol.

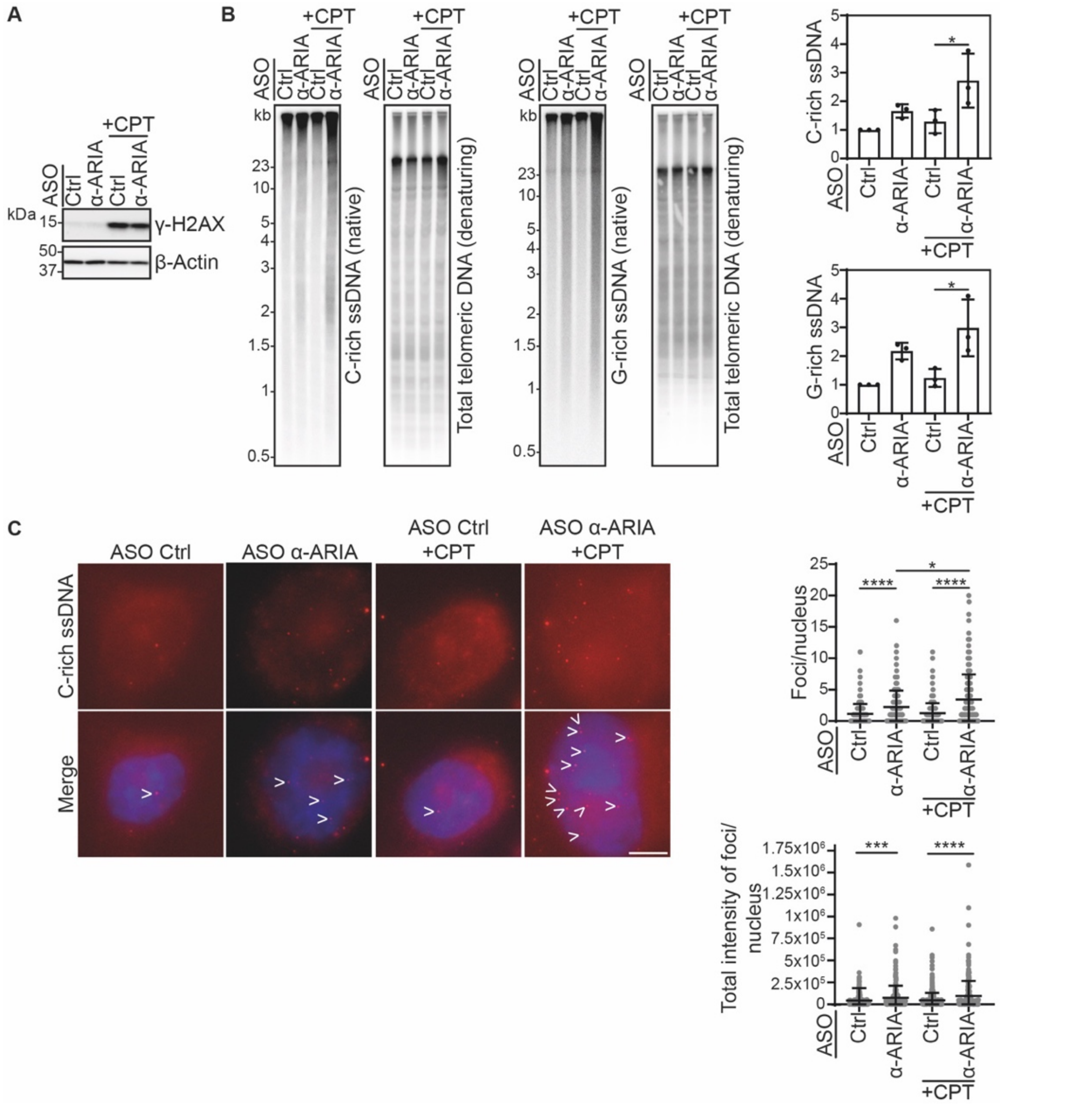

Supplementary Figure S2: Single-stranded DNA accumulation at ITSs in ARIA-depleted CHO cells treated with camptothecin. (**A**) Western blot detecting γ-H2AX and β-Actin (loading control) using total protein extracts from cells transfected with the indicated ASOs and treated with 150 nM camptothecin (CPT) for 1 hour. Marker molecular weights are shown in kDa. (**B**) In-gel hybridizations detecting telomeric repeats using total genomic DNA from cells as in **A**. Gels were first hybridized under native conditions with strand-specific probes to detect single-stranded C-rich and G-rich telomeric repeats. Gels were then denatured and re-probed to detect total telomeric DNA. Molecular weight markers are shown in kb. The graphs on the right are quantifications of ssDNA normalized to the corresponding total DNA and expressed as fold increase over ASO Ctrl samples. Bars and error bars are means and standard deviations from three independent experiments. *P* values were calculated using an ordinary one-way ANOVA followed by Tukey's multiple comparisons test. (**C**) Examples of native DNA FISH detecting C-rich telomeric ssDNA in cells as in **A**. Telomeric DNA is shown in red, DAPI-stained DNA in blue. Arrows point to C-rich telomeric ssDNA. Scale bar: 5 μm. The graphs on the right are quantifications of the numbers and total intensity of telomeric ssDNA foci per nucleus from three independent experiments. At least 63 nuclei were scored per sample in each experiment for a total of at least 249 nuclei. Each dot represents one nucleus. Means and standard deviations are indicated. *P* values were calculated using a Kruskal-Wallis followed by Dunn's multiple comparisons test.

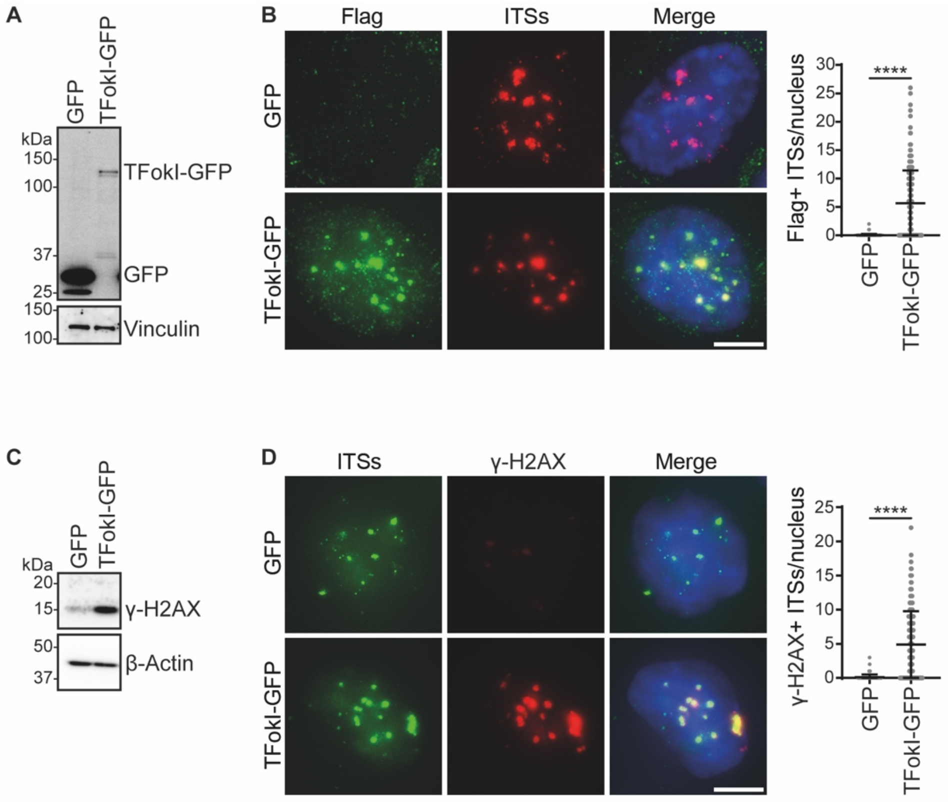

Supplementary Figure S3: Validation of the TRF1-FokI system in CHO cells. (**A**) Western blot detecting GFP using total protein extracts from cells infected with lentiviruses expressing TRF1-FokI-GFP (TFokI-GFP) or GFP only. The membrane was stripped and probed to detect Vinculin as a loading control. Marker molecular weights are shown in kDa. (**B**) Examples of IF/DNA FISH detecting the Flag epitope and telomeric DNA in cells as in **A**. Flag staining is shown in green, telomeric DNA (ITSs) in red, DAPI-stained DNA in blue. Scale bar: 5 μm. The plot on the right is a quantification of the numbers of ITSs staining positive for the Flag epitope (Flag+) per nucleus from three independent experiments. At least 100 nuclei were scored per sample in each experiment. Each dot represents one nucleus. Means and standard deviations are indicated. *P* values were calculated using a two-tailed Mann-Whitney *U* test. (**C**) Western blot detecting γ-H2AX and β-Actin (loading control) using total protein extracts from cells as in **A**. Marker molecular weights are shown in kDa. (**D**) Examples of IF/DNA FISH detecting γ-H2AX and telomeric DNA in cells as in **A**. ITSs are shown in green, γ-H2AX in red, DAPI-stained DNA in blue. Scale bar: 5 μm. The plot on the right is a quantification of the numbers of ITSs staining positive for γ-H2AX (γ-H2AX+) per nucleus from three independent experiments. At least 90 nuclei were scored per sample in each experiment. Each dot represents one nucleus. Means and standard deviations are indicated. *P* values were calculated using a two-tailed Mann-Whitney *U* test.

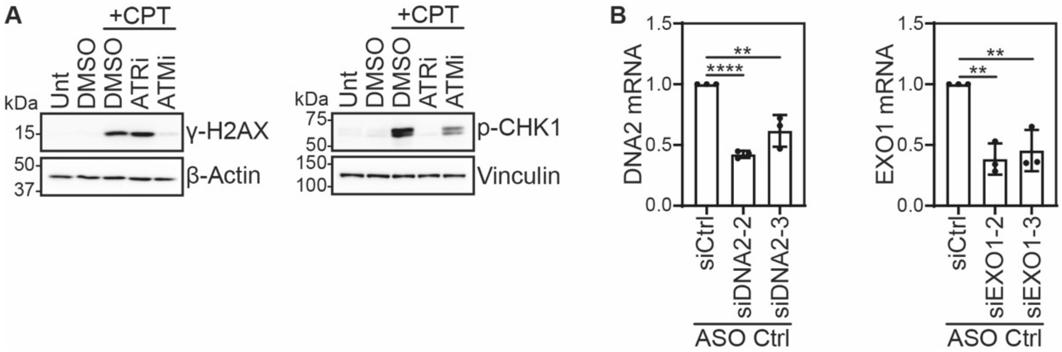

Supplementary Figure S4: Validation of ATM and ATR inhibitors and of DNA2 and EXO1 siRNAs. (**A**) Western blots detecting γ-H2AX and phosphorylated CHK1 (p-CHK1) using total protein extracts from cells treated with 1 μM CPT for 1 hour on top of treatment with DMSO (vehicle), an ATR inhibitor (ATRi) or an ATM inhibitor (ATMi) for 5 hours before harvesting. β-Actin and Vinculin were used as loading controls. Marker molecular weights are shown in kDa. (**B**) Quantifications of RT-qPCRs for DNA2 and EXO1 mRNAs in cells transfected with two independent siRNAs against DNA2 (siDNA2-2 and siDNA2-3), two independent siRNAs against EXO1 (siEXO1-2 and siEXO1-3) or a non-targeting siRNA control (siCtrl). Cells were harvested 72 hours after transfection. GAPDH mRNA was quantified to normalize DNA2 and EXO1. Values are plotted as fold changes over siCtrl samples. Bars and error bars are means and standard deviations from three independent experiments. *P* values were calculated using an unpaired two-tailed Student's *t*-test.

Uncropped blots

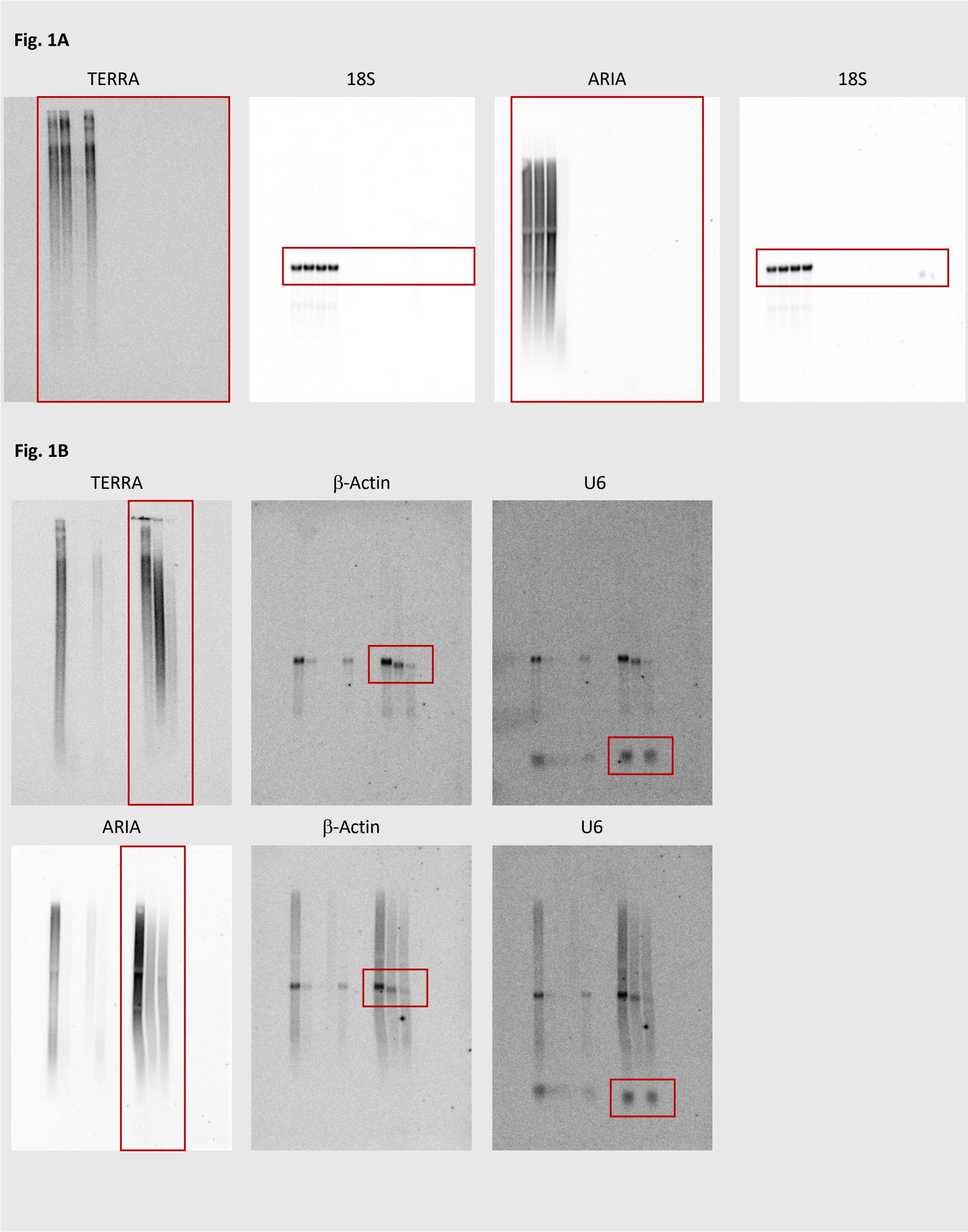

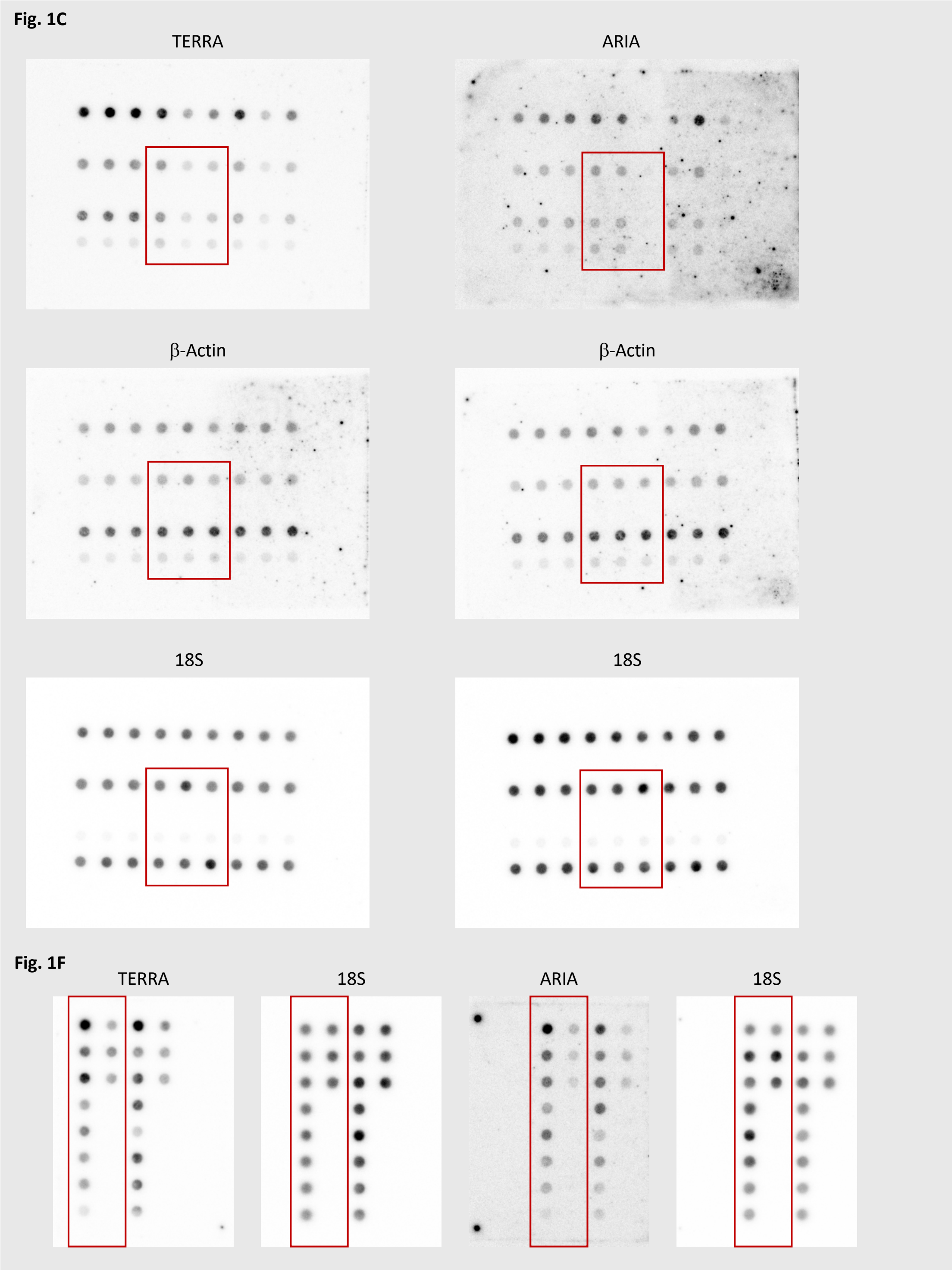
Fig. 1C
TERRA
ARIA
β-Actin
β-Actin
18S
18S
Fig. 1F
TERRA
18S
ARIA
18S

Fig. 3B

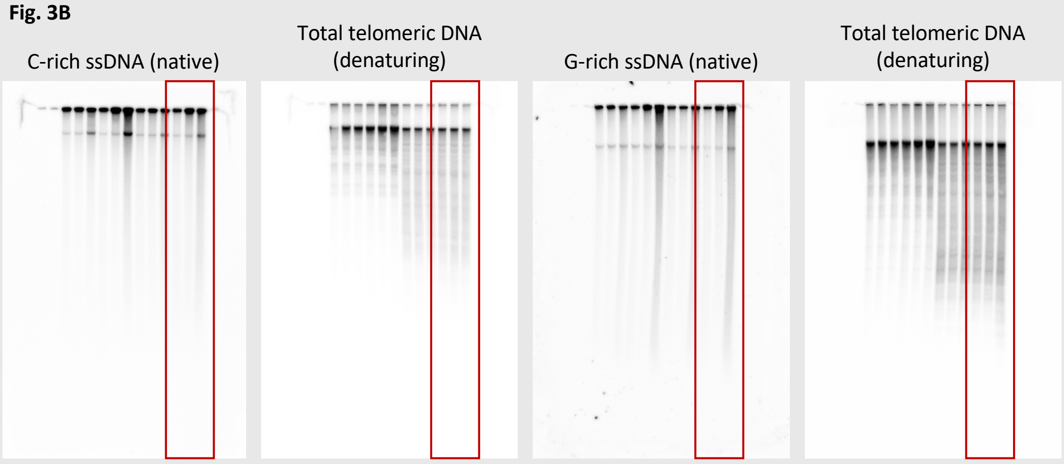
C-rich ssDNA (native)
Total telomeric DNA (denaturing)
G-rich ssDNA (native)
Total telomeric DNA (denaturing)

Fig. 4A

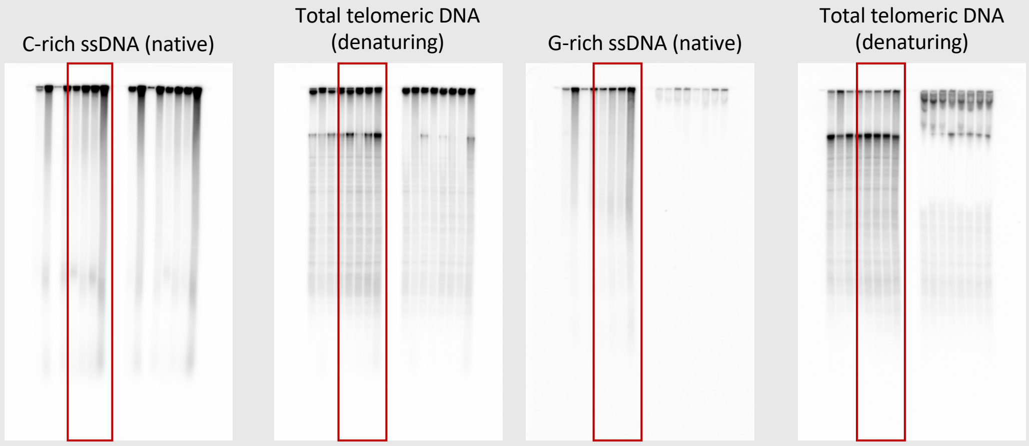
C-rich ssDNA (native)
Total telomeric DNA (denaturing)
G-rich ssDNA (native)
Total telomeric DNA (denaturing)

Fig. S2A

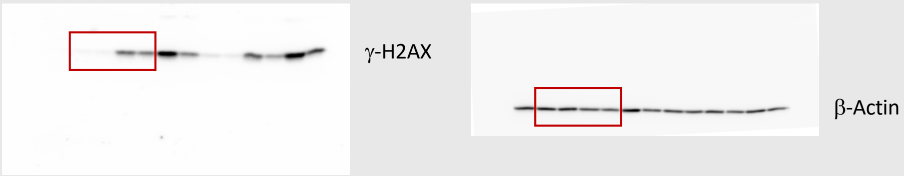

Fig. S2B

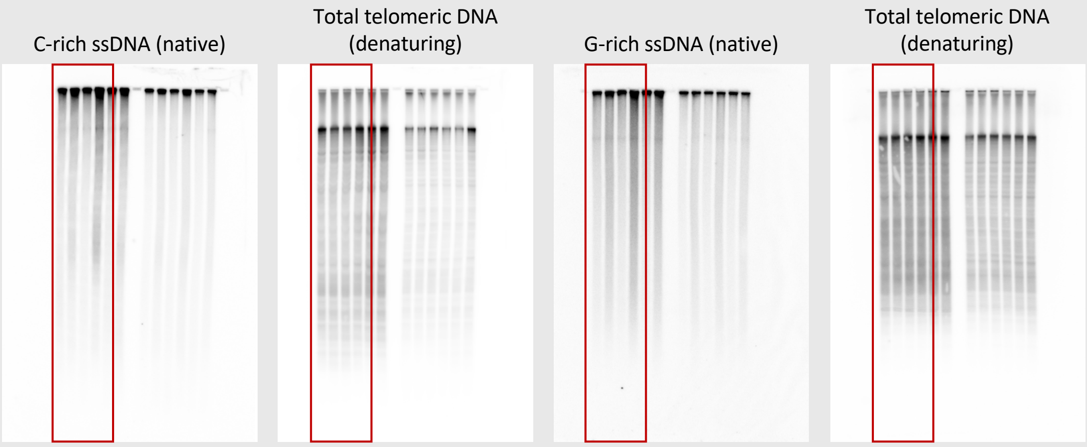

Fig. S3A

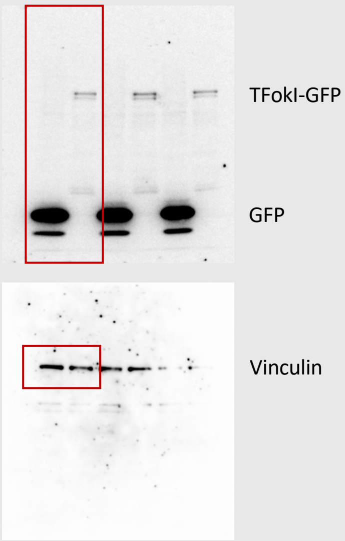

Fig. S3C

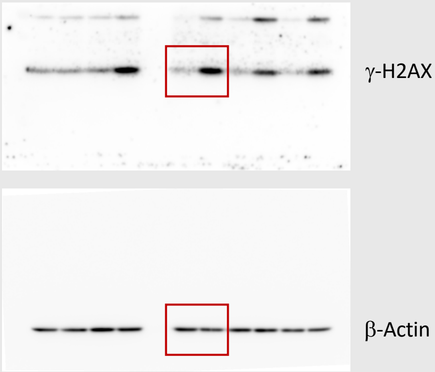

Fig. S4A

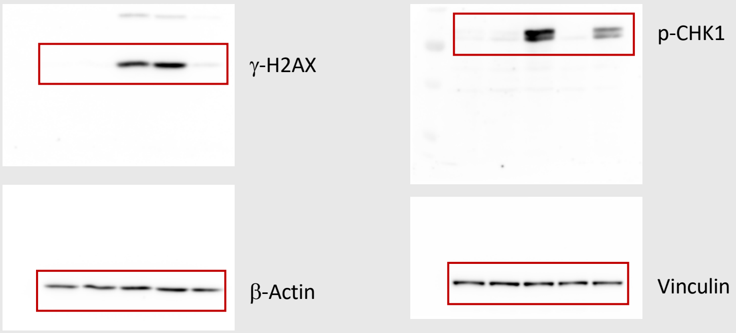

Supplement: gkag739_Supplemental_File [file gkag739_supplemental_file.pdf]
